# Reconstruction of Chronic, Retracted Pectoralis Major Tendon Tear With Achilles Tendon Allograft

**DOI:** 10.1016/j.eats.2022.11.027

**Published:** 2023-03-03

**Authors:** Paolo Miguel Rivera, Dineysh Dhytadak, Gregory Cunningham

**Affiliations:** a Centre Epaule Coude la Colline, Geneva, Switzerland; b MGM Medical College, Aurangabad, Maharashtra, India; c Geneva University Hospitals, Geneva, Switzerland; d Faculty of Medicine, Geneva, Switzerland

## Abstract

Rupture of pectoralis major tendon (PMT) is an uncommon injury, but its incidence has been increasing in the past 2 decades. Although open repair of the torn tendon is the preferred choice of treatment in acute and chronic cases, this often is not possible for chronic retracted tendon injuries. While several techniques have been described for PMT reconstruction, these allografts and autografts are often smaller and less thick than the native PMT. In this study, we describe the use of the Achilles tendon allograft with unicortical suture buttons for the reconstruction of a chronic and retracted PMT. Furthermore, the advantages and disadvantages of this technique are discussed.

## Introduction

The pectoralis major tendon has 2 heads originating from sternum and clavicle while they insert on the lateral lip of bicipital groove on the humerus shaft in a triangular shape. The fibers of individual head twist from their origin before inserting as a single tendon at the bicipital groove. Although the main function of pectoralis major is adduction, the muscle is also involved in internal rotation, extension and forward flexion.^1^

There has been an increasing incidence of reported pectoralis major tears (PMT) in the last 20 years, likely due to an increased interest in weight training and athletics.^2^ A 40% increase in incidence of this type of injury among NFL players in the last 22 years has been reported.^3^ The typical mechanism involved in rupture of the pectoralis major is extension and external rotation of the muscle along with maximal contraction of muscle. According to the classification by Tietjen, the tear can be termed as partial or complete, based on the extent of injury to the 2 tendon layers in the anterior-posterior direction.^4^ Although there is no accepted standardized definition, a systemic review by Elmaraghy et al. defined chronic tears to be occurring after 6 weeks from injury.^5^ The same authors also reported that out of the 287 patients, 62% were acute injuries, and 38% were chronic.^5^

The treatment of PMT depends on the extent of the injury and physical demands of the patient.^2^ Conservative line of management is indicated for contusions, partial tears, muscle intramuscular tears, and complete tears in low-demand patients.^2^ Although the PM is not required for most activities in daily living, surgical management is indicated in the young and athletic patients to avoid loss of strength of muscle during adduction, forward flexion and internal rotation of the joint.^2^

Chronic injuries are associated to adhesions, muscle retraction, possible compromised length-tension relationship during repair and overall unpredictable healing rate.^3^ A recent meta-analysis by Bodendorfer et al. reported that, for chronic cases, operative repair provided better functional outcome, isokinetic/isometric strength, cosmesis, and resting deformity compared to nonoperative management. The authors also concluded that for nonrepairable tears, reconstruction with graft augmentation appears to provide better isometric strength compared to nonoperative management.^2^

For reconstruction with graft augmentation, several authors have described various techniques with the use of the following grafts: semitendinosus and gracilis autografts,^6^^,^^7^^,^^8^ fascia lata allograft,^10^^,^^11^ dermal allografts,11,12,13 bone-patellar bone-tendon autograft,14 and Achilles tendon allograft.^15^

Although no comparative studies have proven the superiority of one over the other, the aim of this Technical Note is to demonstrate the author’s preferred repair technique for chronic PMT using Tendo Achilles allograft supplemented with unicortical button fixation.

## Surgical Technique

See Video 1 under Supplemental Data for the surgical technique. The patient is placed in the beach chair position with an interscalenic bloc and under general anesthesia to ensure maximal muscle relaxation. The arm is fully prepped, draped, and placed on a Trimano positioning system (Arthrex, Naples, FL).

**Fig 1:**
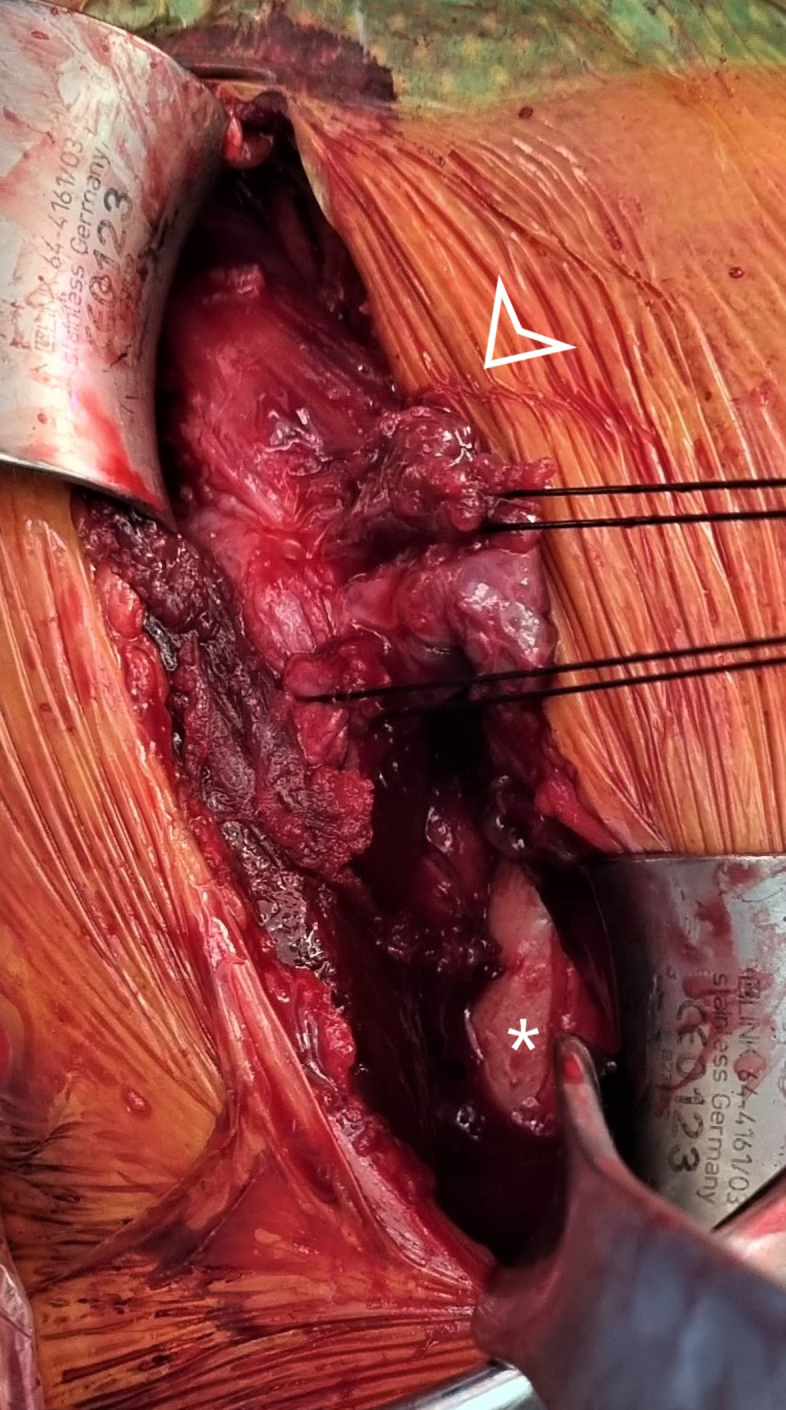
The pectoralis major tendon stump (arrow head) is identified 4 cm medial to the insertion site (asterisk). Traction sutures are applied with Vicryl sutures.

A 9-cm oblique incision is carried out over the pectoralis major insertion site. Deep dissection was carried out until the clavipectoral fascia was identified, which was subsequently excised to allow for better visualization by retracting the deltoid laterally (Fig 1).

The stump of the retracted tendon is identified 3-4 cm medial to the medial lip of its insertion and is held with Kocher clamps for easy manipulation. Further extensive dissection is carried out to free the muscle from the scar tissue and allow for better mobilization of the tendon. It is advised to carefully bluntly dissect the posterior and medial aspect of the muscle to injury to the neurovascular bundles.

**Fig 2:**
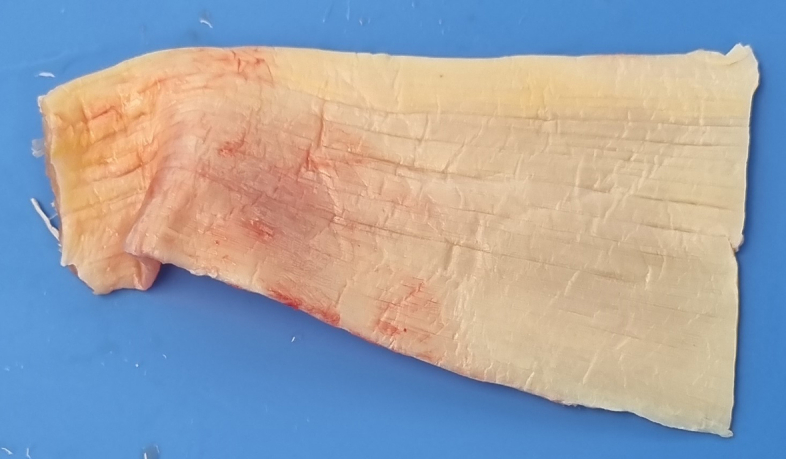
A 6 × 5 cm Achilles tendon allograft is trimmed to a trapezoidal shape to mimic the native pectoralis major tendon.

**Fig 3:**
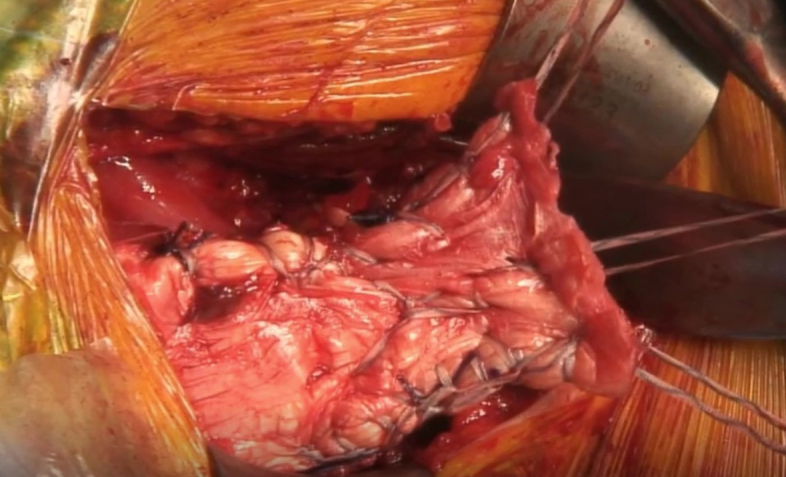
The graft is sutured to the native tendon stump using Krackow stitches in the superior, middle, and inferior aspect of the graft with no. 2 FiberWire sutures (Arthrex, Naples, Florida) to create 3 pairs of suture limbs.

Traction sutures are applied on the tendon with Vicryl 2.0. While in traction, the gap between the tendon and bone insertion site is measured with the arm in neutral rotation. A fresh-frozen Achilles tendon allograft is thawed and trimmed to a trapezoidal shape to reproduce the anatomic shape of the pectoralis major tendon and cover the gap to the insertion site (Fig 2). While in traction, the graft is overlayed on the musculotendinous surface, and its borders are stitched with Vicryl 2.0 mattress sutures. The graft is then whipstitched to the pectoralis major with 3 no. 2 FiberWire sutures (Arthrex, Naples, FL) in a horizontal Krackow fashion, in the superior, middle, and inferior aspect (Fig 3). The sutures should enter and exit the graft posteriorly, and 5-10 mm from its lateral border. Each pair of suture limbs is loaded onto a 2.9 × 10.9 mm Pec Button (Arthrex).

**Fig 4:**
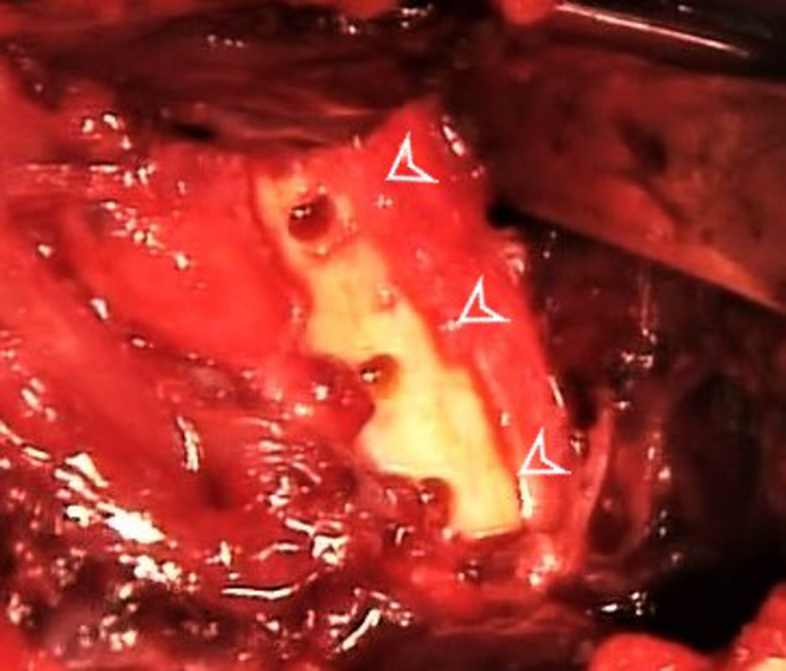
A 3.2-mm drill bit is used to create 3 unicortical holes (arrowheads), respecting the same vertical distance between the 3 pairs of suture limbs.

A curette is used to decorticate the insertion site and 3 unicortical holes are drilled with a 3.2-mm bit, respecting the same vertical distance than between the 3 pairs of sutures. Adequate bone bridge between the holes is recommended in order to prevent the risk of fracture (Fig 4).

**Fig 5:**
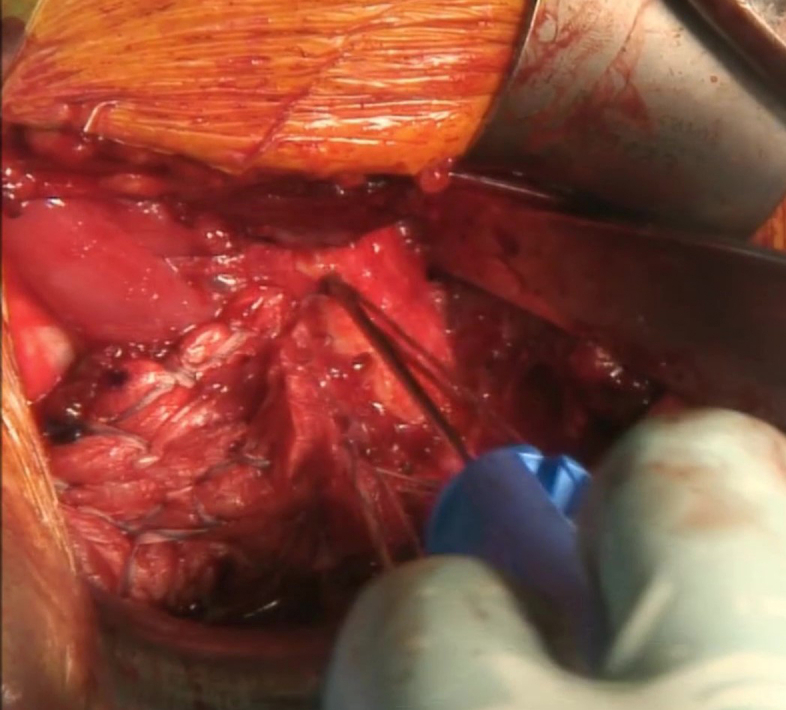
Each pair of sutures are loaded onto a 2.9 × 10.9 mm Pec button (Arthrex). The Pec Button is inserted into its corresponding hole and flipped. Sutures are sequentially tensioned to reduce the graft to the bone.

Each Pec button is inserted into its corresponding hole (Fig 5). The buttons are flipped, and the sutures are sequentially tensioned to reduce the graft to the bone. The sutures are then tied with a standard surgeon’s knot with a total of 7 keys.

**Fig 6:**
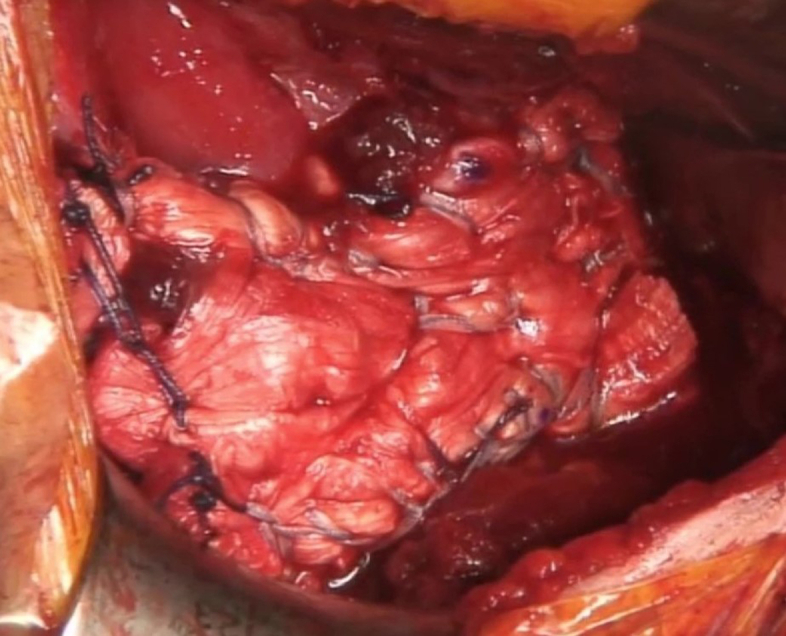
Image showing the final repair construct. Vicryl 2.0 sutures are used to secure the medial aspect of the graft to the native tendon.

The stability of the final construct was tested with internal and external rotation of the arm (Fig 6).

### Postoperative Rehabilitation

The patient is placed in a sling for 4 weeks, and passive range of motion is allowed with no external rotation or extension for the for the first 3 weeks. Gradual strengthening exercises with elastic bands are initiated after 6 weeks. Patients are allowed to go back to sports involving the upper limbs after 3 months.

## Discussion

Although there are numerous studies describing the use of different grafts for pectoralis major reconstruction,^6^, ^7^, ^8^, ^9^, ^10^, ^11^, ^12^, ^13^, ^14^, ^15^ most of them use grafts that are narrower and/or thinner, requiring different construct configurations for the reconstruction procedure. The authors prefer using Achilles tendon allograft for several reasons listed in Table 1. First, its size is similar to the size of the native pectoralis major, which measures ∼5 to 6 cm in medial-lateral length and 4 to 5 cm in proximal-distal width.^1^ A second advantage is that the allograft can be trimmed down to the replicate the individual shape of the pectoralis major tendon insertion. Although there are no biomechanical studies comparing the different grafts for PMT reconstruction, Achilles tendon allograft is a stronger graft. A biomechanical study by Hangody described that without gamma irradiation, the Achilles tendon allograft displayed a higher maximal load compared to the semitendinosis and gracilis allograft. On the other hand, Achilles tendon exhibited a lower young’s modulus and higher strain at tensile strength and at break.^15^ Lennard Funk reported clinical results in unrepairable PMT using tendo-Achilles allograft and suture anchors. The preliminary outcome showed improvement of strength in 82% of patients.^16^ However, the authors believe that unicortical button fixation technique allows solid anchorage of the tendon to the bone, maximizing bone contact because of the small drill size, while allowing optimal sequential graft tensioning. A biomechanical study by Rabuck et al., comparing 3 methods to repair the PMT, showed that the load to failure was higher in cortical button repairs (494 N) compared to suture anchor repairs (383 N).^17^ Another advantage with the use of the Pec Button is that the implant can be evaluated on postoperative radiographs.

**Table 1:** Pros and Cons

**Pros**
Achilles tendon offers larger graft sizes than gracilis or semitendinosus, allowing stronger fixation to the native musculotendinous unit.
Achilles tendon is biomechanically stronger.
Cortical buttons are biomechanically stronger than suture anchors. Cortical buttons can be evaluated on postoperative radiographs. Avoids prolonged operative time and donor site morbidity of an autograft
**Cons**
Added costs and limited availability of allograft
Concern for infection and host rejection Potential metal artifact on postoperative MRI from titanium Pec Button

The limitations of using allograft are the added expense, limited availability, and concern for integration and infection. However, although there are no reports of host rejection and infection with the use of Achilles tendon allograft in PM reconstruction, for ACL reconstruction, there is a low risk (0.1-1.7%) of viral and bacterial transmission from allograft tissues.^18^ In line with this, the use of allografts avoids donor site morbidity (i.e., for fascia lata autograft in this particular setting), and in some situations, such as ACL repair, has been proven to be more cost-effective than patellar autograft (lower morbidity and lesser operative).^19^ The cost effectiveness of allograft use for PMT reconstruction remains yet to be investigated. The limitation of using Pec Button could be the metallic artifacts generated for future MRI analysis. However, recent correction sequences such as SEMAC or MAVRIC could overcome this issue.^20^

## Supplementary Data

ICMJE author disclosure forms

Video 1 Pectoralis major tendon reconstruction using an Achilles tendon allograft. In this video, the following steps are described: preparing the allograft, securing the graft to the native tendon, and securing the graft to the native footprint with cortical buttons. Preliminary clinical and radiographic outcome at 6 weeks are also shown.
